# A Phytase Characterized by Relatively High pH Tolerance and Thermostability from the Shiitake Mushroom *Lentinus edodes*

**DOI:** 10.1155/2013/540239

**Published:** 2013-03-21

**Authors:** Guo-Qing Zhang, Ying-Ying Wu, Tzi-Bun Ng, Qing-Jun Chen, He-Xiang Wang

**Affiliations:** ^1^College of Biosciences and Biotechnology, Beijing University of Agriculture, Beijing 102206, China; ^2^State Key Laboratory of Agro-Biotechnology and MOA Key Laboratory of Soil Microbiology, College of Biological Sciences, China Agricultural University, Beijing 100193, China; ^3^School of Biomedical Sciences, Faculty of Medicine, The Chinese University of Hong Kong, Shatin, New Territories, Hong Kong

## Abstract

A monomeric phytase with a molecular mass of 14 kDa was acquired from fresh fruiting bodies of the shiitake mushroom *Lentinus edodes*. The isolation procedure involved chromatography on DEAE-cellulose, CM-cellulose, Q-Sepharose, Affi-gel blue gel, and a final fast protein liquid chromatography-gel filtration on Superdex 75. The purified phytase demonstrated the unique N-terminal amino acid sequence DPKRTDQVN, which exhibited no sequence similarity with those of other phytases previously reported. It expressed its maximal activity at pH 5.0 and 37°C. Phytase activity manifested less than 20% change in activity over the pH range of 3.0–9.0, considerable thermostability with more than 60% residual activity at 70°C, and about 40% residual activity at 95°C. It displayed a wide substrate specificity on a variety of phosphorylated compounds with the following ranking: ATP > fructose-6-phosphate > AMP > glucose-6-phosphate > ADP > sodium phytate > **β**-glycerophosphate. The phytase activity was moderately stimulated by Ca^2+^, but inhibited by Al^3+^, Mn^2+^, Zn^2+^, and Cu^2+^ at a tested concentration of 5 mM.

## 1. Introduction

Phytic acid (*myo*-inositol 1, 2, 3, 4, 5, 6-hexakisphosphoric acid) is the primary storage form of phosphate in plants and the prime concern for human nutrition and health management [1, 2]. As an antinutritional component, phytic acid can strongly chelate with cations such as calcium, kalium, magnesium, iron, copper, zinc, and as well as proteins [3]. That is why phytic acid adversely affects the mineral absorption and digestion [4]. Phytate (*myo*-inositol 1, 2, 3, 4, 5, 6-hexakisphosphates, phytic acid) generally presented as salt of the mono- or divalent cations as K^+^, Ca^2+^, and Mg^2+^ and is widely distributed in plants, microorganisms, and some animals, and especially in seeds and nuts of plants. Although it is a main storage form of phosphate, phytate cannot be utilized by monogastric animals such as pig, poultry, and humans as they lack phytate-degrading enzymes [5].

The enzyme phytase, chemically known as *myo*-inositol 1, 2, 3, 4, 5, 6-hexakisphosphate phosphohydrolase, belongs to a subclass of the family of histidine acid phosphatases (HAP). It can catalyze the sequential release of phosphate from phytate [6]. Although the first phytase was reported from rice brans in early 20th century, these enzymes are widely distributed among plant, bacteria, yeast, and fungi [7, 8]. Extensive studies conducted on microbial phytases have proved their efficacy which could be tapped for animal nutrition, human health, and environment protection purposes [9, 10]. The first commercial phytase was produced from *Aspergillus niger* and was released to the market in 1991 [1]. At the end of the 20th century, the annual sale of phytase as an animal feed supplement was estimated to be about $500 million [11]. Moreover, its potential in human nutritional improvement and in aquaculture has also being extensively explored [12, 13].

*Lentinus edodes*, commonly referred to as shiitake mushroom, is a popular edible and medicinal mushroom, which is cultivated and consumed in many Asian countries. Compounds isolated from its fruiting bodies or mycelia, such as lectin, laccase, and polysaccharide, have been demonstrated to show a variety of therapeutic properties, especially antitumor, antivirus, and immunomodulatory activities [14–16]. The present investigation was undertaken with an aim to isolate and characterize the phytase from *L. edodes*. The study would supplement the nutritional literature pertaining to this mushroom. 

## 2. Materials and Methods

### 2.1. Materials and Reagents

Fresh fruiting bodies of shiitake mushroom *L. edodes *were purchased from a local market in Beijing, China. DEAE-cellulose, CM-cellulose, Tris-base, sodium phytate, AMP, ADP, ATP, fructose-6-phosphate (F-6-P), glucose-6-phosphate (G-6-P), and *β*-glycerophosphate were obtained from Sigma, St. Louis, MO, USA. Affi-gel Blue gel was purchased from Bio-Rad, Richmond, CA, USA. Q-Sepharose, Superdex 75 HR 10/30 column, and molecular mass standards were obtained from GE Healthcare, USA. All other reagents used were of reagent grade from China unless otherwise mentioned.

### 2.2. Enzyme Assay

Phytase activity was measured using a modified ferrous sulfate-molybdenum blue assay [2]. In brief, 25 *μ*L enzyme solution was incubated with 475 *μ*L of 5 mM sodium phytate in 50 mM Tris-HCl buffer (pH 7.0) at 37°C for 15 min. The enzyme reaction was subsequently terminated by the addition of 500 *μ*L 10% (w/v) trichloroacetic acid. The released phosphate was measured at 700 nm after adding 1000 *μ*L of freshly prepared color reagent, which was composed of 1% (w/v) ammonium molybdate, 3.2% (v/v) sulfuric acid solution, and 7.2% (w/v) ferrous sulfate solution. One unit of phytase activity was defined as the amount of enzyme needed to liberate 1 *μ*mol phosphate per min under the assay conditions. Protein was determined according to Bradford using a protein assay kit (Bio-Rad Lab, Richmind, CA, USA) with bovine serum albumin as the standard [17]. All determinations were performed in triplicate.

### 2.3. Purification of Phytase

Fresh fruiting bodies of shiitake mushroom (2000 g) were homogenized and extracted with cold distilled water (4 mL/g) at 4°C for 4 h. Following the centrifugation at 12000 rpm for 15 min, the supernatant obtained was subjected to ultrafiltration until the volume was reduced to 100 mL. NH_4_HCO_3_ buffer (1 M, pH 9.5) was added until the molarity of NH_4_HCO_3_ attained 10 mM. Ion exchange chromatography on a DEAE-cellulose column (2.5 cm × 30 cm) was conducted in 10 mM NH_4_HCO_3_ buffer (pH 9.5). After the removal of unadsorbed materials (fractions D1 and D2), the column was washed with 1 M NaCl in the 10 mM NH_4_HCO_3_ buffer (pH 9.5) to remove adsorbed materials. Fraction D2 enriched in phytase activity was dialyzed against 10 mM NH_4_OAc buffer and then subjected to ion exchange chromatography on a 2.5 cm × 20 cm column of CM cellulose in 10 mM NH_4_OAc buffer (pH 4.5). The unadsorbed fraction CM1 containing phytase activity was collected before the desorption of inactive adsorbed materials (collected as fraction CM2) with 10 mM NH_4_OAc buffer (pH 4.5) containing 1 M NaCl. Fraction CM1 was then applied on a 2.5 cm × 20 cm column of Affi-gel Blue gel in 10 mM Tris-HCl buffer (pH 7.5). Unadsorbed fraction with phytase activity (fraction B1) was eluted with the same buffer while adsorbed proteins devoid of phytase activity (fraction B2) were desorbed with 1 M NaCl added to the 10 mM Tris-HCl buffer. Fraction B1 was subsequently chromatographed on a 1.0 cm × 30 cm column of Q-Sepharose in 10 mM NH_4_HCO_3_ buffer (pH 9.5). Unadsorbed proteins were eluted into two fractions, Q1 and Q2, while adsorbed proteins were eluted into fraction Q3 with 1 M NaCl in the starting buffer 10 mM NH_4_HCO_3_ (pH 9.5). Fraction Q2 with phytase activity was next subjected to gel filtration by fast protein liquid chromatography (FPLC) on a Superdex 75 HR 10/30 column in 0.2 M NH_4_HCO_3_ buffer (pH 8.5) using an AKTA Purifier system (GE Healthcare, USA). The second peak (SU2) represented purified phytase.

### 2.4. Determination of Molecular Mass

The molecular mass of the purified phytase was determined using sodium dodecyl sulfate polyacrylamide gel electrophoresis (SDS-PAGE) and FPLC-gel filtration. In SDS-PAGE, a 12% resolving gel and a 5% stacking gel were used, with a procedure as described by Laemmli and Favre [23]. At the end of electrophoresis, the gel was stained with 0.1% Coomassie brilliant blue G-250. The molecular mass of the purified phytase was calculated using a lg*Mr* migrate rate curve based on the molecular mass standards used in SDS-PAGE. In FPLC-gel filtration, another curve of elution volume-lg*Mr* was obtained. The molecular mass of purified phytase was calculated using the curve and its elution volume.

### 2.5. Determination of N-Terminal Amino Acid Sequence

The N-terminal sequence of the phytase was determined and carried out using an HP G1000A Edman degradation unit and an HP 1000 HPLC system.

### 2.6. Assay for pH Optimum, Temperature Optimum, and Thermostability of Purified Phytase

A series of sodium phytate solution in buffers with different pH values including 50 mM NaOAc (pH 3.0–5.0), 50 mM Mes (pH 5.0–7.0), and 50 mM Hepes (pH 7.0–9.0) were used to determine the optimal pH value. For determining the optimal temperature, the reaction mixture was incubated at 20°C, 30°C, 37°C, 45°C, 50°C, 60°C, 70°C, 80°C, and 100°C in 50 mM NaOAc (pH 5.0) for 15 min, respectively. In the thermostability assay, enzyme solutions were previously incubated at various temperatures (45°C, 60°C, 70°C, and 80°C) for various durations (10, 20, 40, and 60 min), respectively. The residual activity was measured using the standard assay after the enzyme solutions had been cooled down to room temperature. All determinations were performed in triplicate.

### 2.7. Assay for Substrate Specificity

In order to determine the substrate specificity of the purified phytase, several phosphorylated substrates instead of sodium phytate, all at 5 mM concentrations, were added to the assay solution. They included AMP, ADP, ATP, fructose-6-phosphate, glucose-6-phosphate, and *β*-glycerophosphate. The buffer used was 50 mM NaOAc (pH 5.0). The release of Pi was determined as mentioned above.

### 2.8. Effects of Metal Ions and EDTA on Phytase Activity

Equal volumes (25 *μ*L) of metal ions or EDTA (with a final concentration of 1 mM and 5 mM) were mixed with the purified phytase solution in 50 mM NaOAc buffer (pH 5.0) for 2 h at 4°C before the standard phytase assay was performed. The activity assayed in the absence of metal ions was defined as the control. The metal ions tested include K^+^, Ca^2+^, Mg^2+^, Mn^2+^, Zn^2+^, Cu^2+^, Fe^3+^, and Al^3+^.

## 3. Results

### 3.1. Isolation of Phytase

**Figure 1:**
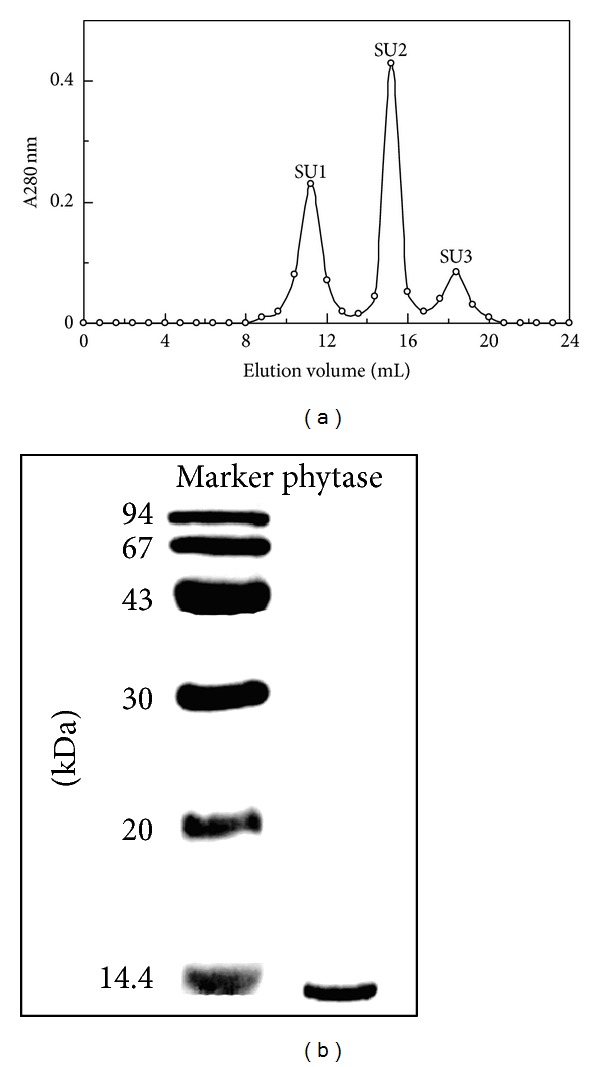
(a) FPLC-gel filtration on Superdex 75 HR 10/30 column. Eluent: 0.2 M NH_4_HCO_3_ buffer (pH 8.5). Fraction size: 0.8 mL. Flow rate: 0.4 mL/min. Fraction SU2 represents purified phytase. (b) SDS-PAGE of fraction SU2.

*L. edodes* phytase was purified by utilizing an isolation protocol that included one step of extraction with distilled water, three unsuccessive steps of ion-exchange chromatography on DEAE-cellulose, CM-cellulose, and Q-Sepharose, an affinity chromatography step on, and one final step of FPLC on a Superdex 75 column. Chromatographic details including yield, recovery rate, and purification fold are presented in Table 1. *L. edodes* phytase was adsorbed neither on the three ion-exchange gel nor on Affi-gel blue gel. The fraction with phytase activity from the penultimate step was finally separated into three peaks on Superdex 75 (Figure 1(a)). The second and highest peak (SU2) was the purified phytase with a molecular mass of 14 kDa based on its elution volume on FPLC-gel filtration. The enzyme was purified 34.6-fold from the crude extract with 20.7% yield. The purified phytase exhibited an activity of 3.11 U/mg. Fraction SU2 subsequently appeared as a single 14 kDa band in SDS-PAGE (Figure 1(b)). Based on the results of FPLC and SDS-PAGE, the purified phytase was a monomeric protein with a molecular mass of 14 kDa.

### 3.2. Properties of *L. edodes* Phytase

**Figure 2:**
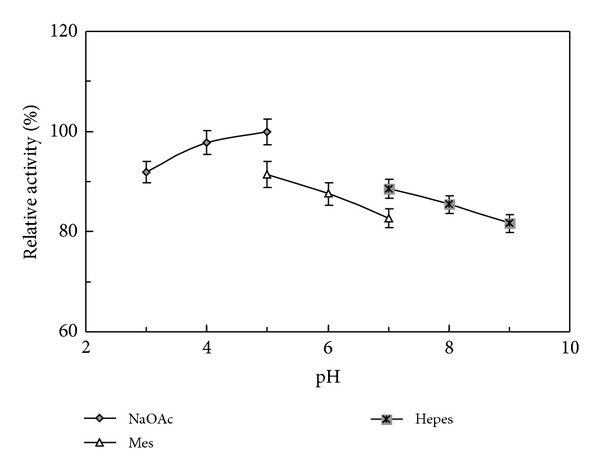
Effect of pH on activity of the purified *L. edodes* phytase. Maximal phytase activity at pH optimum was defined as 100%. Results are presented as mean ± SD (*n* = 3).

**Figure 3:**
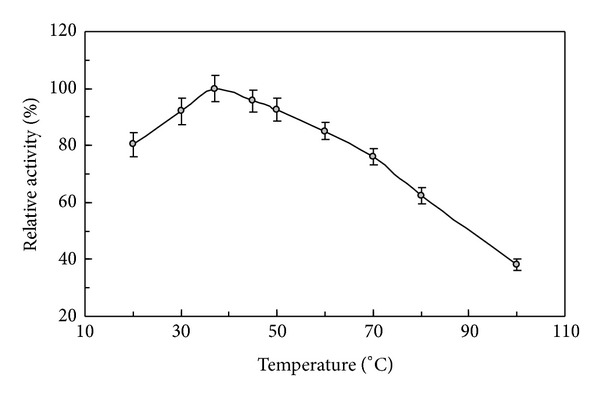
Effect of temperature on activity of the purified *L. edodes* phytase. Maximal phytase activity at temperature optimum was defined as 100%. Results are presented as mean ± SD (*n* = 3).

**Figure 4:**
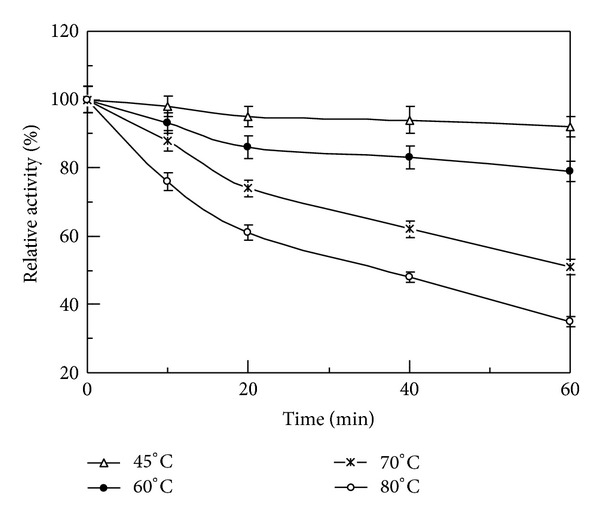
Thermostability of the purified *L. edodes* phytase. Phytase activity at 0 min was defined as 100%. Results are presented as mean ± SD (*n* = 3).

The N-terminal sequence of the purified phytase was DPKRTDQVN. A comparison of the characteristics of *L. edodes* phytase and other fungal phytases is presented in Table 2. The purified phytase expressed its maximal sodium phytate degradative activity at pH 5.0 (Figure 2) and 37°C (Figure 3). It manifested less than 20% fluctuation in activity over the pH range of 3.0–9.0. When the assay temperature was increased from 20°C to 37°C, the phytase reached its maximum degradative activity. It underwent a continuous decline in enzyme activity when the temperature was elevated further from 37°C to 95°C. More than 60% phytase activity remained when it was assayed at 70°C. About 40% residual activity can be measured when it was assayed at 95°C. The purified phytase was fairly thermostable with less than 10% activity loss for 60 min incubation at 45°C, and about 20% activity loss for 60 min incubation at 60°C (Figure 4). *L. edodes* phytase demonstrated a wide substrate specificity on a variety of phosphorylated compounds with the following ranking: ATP > F-6-P > AMP > G-6-P > ADP > sodium phytate > *β*-glycerophosphate (Table 3). The phytase activity was moderately stimulated by Ca^2+^ at a tested concentration of 5 mM, and not significantly affected by K^+^, Ca^2+^, Mg^2+^, Mn^2+^, Cu^2+^, Fe^3+^, and EDTA at a tested concentration of 1 mM, and K^+^, Mg^2+^, Fe^3+^, and EDTA at a tested concentration of 5 mM. Furthermore, the phytase was moderately inhibited by Al^3+^, Mn^2+^, Zn^2+^, and Cu^2+^ at a tested concentration of 5 mM (Table 4).

## 4. Discussion

The shiitake mushroom is the second most popular edible mushroom in the global market and has long been considered a medicinal mushroom as well as a delicacy in Asian countries [24]. It is one of the most nutritious and medicinal mushrooms with potential therapeutic applications involving cancers, flu, heart diseases, hypertension, diabetes, antiageing, obesity, sexual dysfunction, and so on [25]. Phytase is commercially utilized to maximize phytate degradation in diet and to decrease phosphorus levels in poultry and swine manure. Extensive studies on microbial phytases especially from genus *Aspergillus* have been reported involving isolation, properties, fermentation, cloning, expression, structure, and so forth [6, 9, 26]. There is a dearth of literature on mushroom phytases. Collopy and Royse observed that the fruiting bodies of four edible mushroom, *Agaricus bisporus*, *Grifola frondosa*, *Pleurotus cornucopiae*, and *L. edodes*, manifested phytase activity although no details on phytase purification were reported [27]. In the present study, we focused on *L. edodes* phytase which played a very important role in digestion and utilization of dietary phosphorus. 

During the course of isolation,* L. edodes* phytase was unadsorbed on all ion-exchangers and affinity chromatography media employed including DEAE-cellulose, CM-cellulose, Q-Sepharose, and Affi-gel blue gel. Compared with* L. edodes* phytase, *Volvariella volvacea* phytase showed an analogous chromatographic behavior, in wich it was adsorbed on Q-Sepharose, but unadsorbed on DEAE-cellulose, CM-cellulose, and Affi-gel blue gel [19]. On the other hand, *Aspergillus ficcum* phytase was adsorbed on both DEAE-cellulose and CM-cellulose [2]. *Flammulina velutipes* phytase was adsorbed on DEAE-cellulose and Q-Sepharose, but unadsorbed on CM-cellulose and Affi-gel blue gel [18].

*L. edodes* phytase was a monomeric protein with a molecular mass of 14 kDa based on the results of FPLC and SDS-PAGE, which were very similar to phytases from* V. volvacea* (14 kDa) [19] and *F. velutipes* (14.8 kDa) [18]. Molecular masses from phytases of the genus *Aspergillus* fell within the range of 60–120 kDa. Phytases from bacteria such as *Bacillus subtilis* and *Escherichia coli* demonstrated molecular masses in the proximity of 40 kDa. On the other hand, plant phytases showed a wide molecular masses range from 60 kDa (characteristic of Spelt, a kind of wheat) to 3699 kDa (characteristic of *Lilium longiflorum*) [26]. Based on the protein blast search result of the N-terminal amino acid sequence (http://blast.ncbi.nlm.nih.gov/Blast.cgi), *L. edodes* phytase demonstrated no sequence similarity to other phytases. At the same time, it manifested high sequence homology with a nucleoside-diphosphate-sugar epimerase from *Synechococcus* sp. (YP_001227174.1) and a signal transduction histidine kinase from *Kytococcus sedentarius* (YP_003150103.1) both of which are phosphorus transportation enzymes.

The optimal pH for shiitake mushroom phytase was pH 5.0, with less than 20% variation in activity detected in the pH range of 3.0–9.0. The phytase demonstrates considerable pH adaptability compared with other phytases reported. Two kinds of phytases (PhyA and PhyB) were isolated from *A. niger*. *A. niger* PhyA exhibited two pH optima at pH 2.5 and pH 5.0, while *A. niger* PhyB demonstrated only a single pH optimum at pH 2.5 [8]. Another PhyB from *A. ficcum* manifested a pH optimum at pH 1.3, the lowest among all the phytases reported [2]. All three phytases showed highly pH-dependent enzyme activity. *A. ficcum* phytase lost virtually its whole activity when the ambient pH value reached 5.0 [2]. On the other hand, mushroom phytases from* V. volvacea* [19] and *F. velutipes* [18] possessed the same pH optimum at pH 5.0, and also considerably stability over the pH range of 3.0–9.0. According to the results, the three mushroom phytases (*L. edodes*, *V. volvacea*, and *F. velutipes*) manifested a better pH tolerance than commercial *Aspergillus* phytases. Although shared similar characteristics in molecular mass and optimal pH value, the three mushroom phytases showed very low N-terminal amino acid sequence similarity and different chromatography behavior.

The purified phytase required a low temperature optimum of 37°C, which was the same as the body temperature in humans, but lower than those of other phytases reported [26]. Most of them had a temperature optimum in a temperature range of 45–70°C including *V. volvacea* (45°C) [19], *F. velutipes* (45°C) [18], *A. niger* PhyA (58°C) [20], *A. niger* PhyB (60°C) [22], *A. ficcum* PhyB (65°C) [2], and *Schwanniomyces castellii* (77°C) [28]. *L. edodes* phytase also demonstrated considerable thermostability with about 60% residual activity at 70°C and 40% residual activity at 95°C. *A. ficcum* PhyB was relatively stable at 60°C with 14% loss in activity after exposure to the temperature for 60 min, while only about 50% of the highest enzyme activity was retained at 37°C [2]. The purified phytase manifested the desirable features of relative pH tolerance and thermostability, which makes it a promising candidate with more potential applications.

Phytase from shiitake mushroom exhibited broad substrate specificity on a range of phosphorylated compounds. *A. niger* PhyB has been reported to have a wider substrate specificity than *A. niger* phyA. *A. niger* phyA showed the highest activity towards sodium phytate, but very low activity towards other substrates such as F-6-P, G-6-P, and ADP. *A. niger* phyB had an activity toward F-1, 6-P which was 20 times higher than that towards sodium phytate [26]. The enzyme activity of *L. edodes* phytase was the highest towards ATP, like phytases from *A. ficcum* PhyB [2], *V. volvacea* [19], *F. velutipes* [18], and Spelt [26]. **ρ**-nitrophenyl phosphate and F-1, 6-P formed the optimal substrates of *A. fumigatus* phytase, followed by ATP. On the other hand, alkaline phytases from *B. subtilis*, *B. subtilis natto*, *B. amyloliquefaciens*, and *Typa latifolia* had narrow substrate specificity and can use sodium phytate as their sole substrate [26]. 

The phytase activity was moderately stimulated by Ca^2+^ at a concentration of 5 mM, and not significantly affected by K^+^, Ca^2+^, Mg^2+^, Mn^2+^, Cu^2+^, Fe^3+^, and EDTA at a low concentration of 1 mM, but moderately inhibited by Al^3+^, Mn^2+^, Zn^2+^, and Cu^2+^ at a higher concentration of 5 mM. It was previously reported that Ca^2+^ had a moderately inhibitory effect on phytases from *Cladosporium *sp. [29], *A. niger* [30], and *Klebsiella pneumoniae* [31]. On the other hand, Ca^2+^ showed no significant effect on the phytase from *A. ficcum* [2]. Chelating reagents such as EDTA did not have any inhibitory effect at a concentration of 5 mM, just like other phytases from *Candida krusei* [32] and *K. pneumoniae* [31], but different from a bacterial phytate from *Bacillus* sp. which is strongly inhibited by EDTA at a low concentration of 1 mM [33]. On the contrary, 10 mM EDTA stimulated the activity of fungal phytases from *A. ficcum* [2] and *A. fumigatus* [34].

To recapitulate, *L. edodes* phytase displayed some characteristics distinct from those of animal, plant, bacterial, fungal, and mushroom phytases previously reported in the literature. It manifested the advantageous properties of pH tolerance and thermostability. It signifies that the shiitake phytase has a great potential for commercial interest as an animal feed additive or dietary adjuvant.

## Figures and Tables

**Table 1:** Summary of purification procedure of *L. edodes* phytase (from 2000 g fresh fruiting bodies).

Purification step	Yield (mg)	Total activity (U)^a^	Specific activity (U/mg)^b^	Recovery rate (%)	Purification fold^c^
Water extract	4515.0	406.4	0.09	100	1
DEAE cellulose	498.2	303.9	0.61	74.8	6.8
CM cellulose	252.3	219.5	0.87	54.0	9.7
Blue gel	137.8	154.3	1.12	38.0	12.4
Q-sepharose	63.5	112.4	1.77	27.7	19.7
Superdex 75	27.1	84.3	3.11	20.7	34.6

^a^Total activity: phytase activity (U/mL) in each step × volume (mL);

^b^Specific activity: total activity/yield;

^c^Purification fold: specific activity of each step/specific activity of the first step.

**Table 2:** Characteristics of *L. edodes* phytase with other fungal phytases.

Species	Amino acid sequence	Chromatography behavior	Molecular mass (kDa)	pH optimum	Temperature optimum (°C)
*L. edodes* (this study)	1 DPKRTDQVN 9	Unadsorbed on DEAE-cellulose, CM-cellulose, blue gel, and Q-Sepharose	14	5.0	37
*Flammulina velutipes* [18]	1 **D**FQVDTG**N**N 10	Adsorbed on DEAE-cellulose and Q-Sepharose; unadsorbed on CM-cellulose and Blue gel	14.8	5.0	45
*Volvariella volvacea* [19]	1 GEDNEHDTQA 10	Adsorbed on Q-Sepharose; unadsorbed on DEAE-cellulose, CM-cellulose, and Blue gel	14	5.0	45
*Aspergillus niger* (PhyA) [8, 20, 21] (emb: CAA78904.1)	218 **D**SELA**D**T**V**E 227	—	85	2.5, 5.0	58
*A. niger* (PhyB) [8, 22] (pdb:1QFX)	46 **DP**PTSCEV**DQV**I 57	—	85–100	2.5	60
*A. ficcum* (PhyB) [2]	46 **DP**PTSCEV**DQV**I 57	Adsorbed on DEAE-cellulose and CM-cellulose	65.5	1.3	67
*Kodamaea ohmeri* [1, 2] (ABU53001.1)	24 T**P**EQAAVE**Q**Y**N** 34	Adsorbed on DEAE sepharose	98.2	5.0	65

—: no data available. Sequence analysis using DNAMAN V6.0.3.99.

Amino acid residues identical to corresponding residues of *L. edodes* phytase are underlined.

**Table 3:** Substrate specificity of *L. edodes* phytase.

Substrate	Relative activity (%)
Sodiumm phytate	100.0 ± 5.1
*β*-glycerophosphate	98.5 ± 3.7
ADP	103.4 ± 6.2
G-6-P	127.9 ± 3.9
AMP	143.8 ± 7.0
F-6-P	158.2 ± 4.6
ATP	208.1 ± 6.4

The phytase activity towards sodium phytate (5.0 mM) was regarded as 100%. Phytase activity was assayed with increasing concentration of Pi using the standard phytase assay. Results are presented as mean ± SD (*n* = 3).

**Table 4:** Effects of metal ions and EDTA on phytase activity.

	Relative activity (%)	
	1 mM	5 mM
K^+^	101.5 ± 3.2	104.9 ± 2.8
Ca^2+^	107.6 ± 3.1	114.8 ± 5.3
Mg^2+^	102.7 ± 0.8	103.5 ±3.5
Mn^2+^	95.4 ± 5.1	69.6 ± 2.7
Zn^2+^	90.2 ± 2.1	76.5 ± 1.6
Cu^2+^	104.6 ± 2.5	82.8 ± 2.3
Fe^3+^	96.9 ± 3.0	100.2 ± 1.7
Al^3+^	67.6 ± 1.3	32.5 ± 0.9
EDTA	100.7 ± 2.8	103.4 ± 2.3

The phytase activity in the absence of metal ions was regarded as 100%. Data are given as means ± SD, *n* = 3.

## References

[1] Kumar V, Sinha AK, Makkar HPS, Becker K (2010). Dietary roles of phytate and phytase in human nutrition: a review. *Food Chemistry*.

[2] Zhang GQ, Dong XF, Wang ZH, Zhang Q, Wang HX, Tong JM (2010). Purification, characterization, and cloning of a novel phytase with low pH optimum and strong proteolysis resistance from *Aspergillus ficuum* NTG-23. *Bioresource Technology*.

[3] Luo H, Huang H, Yang P (2007). A novel phytase appA from *Citrobacter amalonaticus* CGMCC 1696: gene cloning and overexpression in Pichia pastoris. *Current Microbiology*.

[4] Raboy V (2003). myo-Inositol-1,2,3,4,5,6-hexakisphosphate. *Phytochemistry*.

[5] Lei XG, Porres JM (2003). Phytase enzymology, applications, and biotechnology. *Biotechnology Letters*.

[6] Haefner S, Knietsch A, Scholten E, Braun J, Lohscheidt M, Zelder O (2005). Biotechnological production and applications of phytases. *Applied Microbiology and Biotechnology*.

[7] Suzuki U, Yoshimura K, Takaishi M (1907). About the enzyme “phytase”, which splits ‘‘anhydro-oxy-methylene diphosphoric acid. *Bulletin of the College of Agriculture, Tokyo Imperial University*.

[8] Mullaney EJ, Daly CB, Ullah AHJ (2000). Advances in phytase research. *Advances in Applied Microbiology*.

[9] Pandey A, Szakacs G, Soccol CR, Rodriguez-Leon JA, Soccol VT (2001). Production, purification and properties of microbial phytases. *Bioresource Technology*.

[10] Vohra A, Satyanarayana T (2003). Phytases: microbial sources, production, purification, and potential biotechnological applications. *Critical Reviews in Biotechnology*.

[11] Abelson PH (1999). A potential phosphate crisis. *Science*.

[12] Yoo GY, Wang X, Choi S, Han K, Kang JC, Bai SC (2005). Dietary microbial phytase increased the phosphorus digestibility in juvenile Korean rockfish *Sebastes schlegeli* fed diets containing soybean meal. *Aquaculture*.

[13] Da Silva LG, Trugo LC, Da Costa Terzi S, Couri S (2005). Low phytate lupin flour based biomass obtained by fermentation with a mutant of *Aspergillus niger*. *Process Biochemistry*.

[14] Tsivileva OM, Nikitina VE, Loshchinina EA (2008). Isolation and characterization of *Lentinus edodes* (Berk.) singer extracellular lectins. *Biochemistry*.

[15] Jeurink PV, Noguera CL, Savelkoul HFJ, Wichers HJ (2008). Immunomodulatory capacity of fungal proteins on the cytokine production of human peripheral blood mononuclear cells. *International Immunopharmacology*.

[16] Rincao VP, Yamamoto KA, Ricardo NM (2012). Polysaccharide and extracts from *Lentinula edodes*: structural features and antiviral activity. *Virology Journal*.

[17] Harlow E, Lane D (2006). Bradford assay. *CSH Protocols*.

[18] Zhu MJ, Wang HX, Ng TB (2011). Purification and identification of a phytase from fruity bodies of the winter mushroom, *Flammulina velutipes*. *African Journal of Biotechnology*.

[19] Xu L, Zhang G, Wang H, Ng TB (2012). Purification and characterization of phytase with a wide pH adaptation from common edible mushroom *Volvariella volvacea* (Straw mushroom). *Indian Journal of Biochemistry & Biophysics*.

[20] Ullah AH (1988). *Aspergillus ficuum* phytase: partial primary structure, substrate selectivity, and kinetic characterization. *Preparative Biochemistry*.

[21] Ullah AH, Gibson DM (1987). Extracellular phytase (E.C. 3.1.3.8) from *Aspergillus ficuum* NRRL 3135: purification and characterization. *Preparative Biochemistry*.

[22] Ullah AHJ, Phillippy BQ (1994). Substrate selectivity in *Aspergillus ficuum* phytase and acid phosphatases using myo-inositol phosphates. *Journal of Agricultural and Food Chemistry*.

[23] Laemmli UK, Favre M (1973). Maturation of the head of bacteriophage T4. I. DNA packaging events. *Journal of Molecular Biology*.

[24] Bisen PS, Baghel RK, Sanodiya BS, Thakur GS, Prasad GBKS (2010). *Lentinus edodes*: a macrofungus with pharmacological activities. *Current Medicinal Chemistry*.

[25] Breene W (1990). Nutritional and medicinal value of specialty mushrooms. *Journal of Food Protection*.

[26] Oh BC, Choi WC, Park S, Kim YO, Oh TK (2004). Biochemical properties and substrate specificities of alkaline and histidine acid phytases. *Applied Microbiology and Biotechnology*.

[27] Collopy PD, Royse DJ (2004). Characterization of phytase activity from cultivated edible mushrooms and their production substrates. *Journal of Agricultural and Food Chemistry*.

[28] Segueilha L, Lambrechts C, Boze H, Moulin G, Galzy P (1992). Purification and properties of the Phytase from *Schwanniomyces castellii*. *Journal of Fermentation and Bioengineering*.

[29] Quan CS, Tian WJ, Fan SD, Kikuchi JI (2004). Purification and properties of a low-molecular-weight phytase from *Cladosporium* sp. FP-1. *Journal of Bioscience and Bioengineering*.

[30] Casey A, Walsh G (2003). Purification and characterization of extracellular phytase from *Aspergillus niger* ATCC 9142. *Bioresource Technology*.

[31] Escobin-Mopera L, Ohtani M, Sekiguchi S (2012). Purification and characterization of phytase from *Klebsiella pneumoniae* 9-3B. *Journal of Bioscience and Bioengineering*.

[32] Quan CS, Fan SD, Zhang LH, Wang YJ, Ohta Y (2002). Purification and properties of a phytase from *Candida krusei* WZ-001. *Journal of Bioscience and Bioengineering*.

[33] Kim YO, Kim HK, Bae KS, Yu JH, Oh TK (1998). Purification and properties of a thermostable phytase from *Bacillus* sp. DS11. *Enzyme and Microbial Technology*.

[34] Wyss M, Brugger R, Kronenberger A (1999). Biochemical characterization of fungal phytases (myo-inositol hexakisphosphate phosphohydrolases): catalytic properties. *Applied and Environmental Microbiology*.

